# Multi-element X-ray movie imaging with a visible-light CMOS camera

**DOI:** 10.1107/S1600577518014273

**Published:** 2019-01-01

**Authors:** Wenyang Zhao, Kenji Sakurai

**Affiliations:** a University of Tsukuba, 1-1-1 Tennodai, Tsukuba, Ibaraki 305-0006, Japan; b National Institute for Materials Science, 1-2-1 Sengen, Tsukuba, Ibaraki 305-0047, Japan

**Keywords:** multi-element X-ray movie imaging, CMOS camera

## Abstract

For many years, X-ray movies have been considered a promising tool for exploring and providing insights into chemical reactions. A simultaneous multi-element X-ray movie can further clarify the behavior difference of various elements and help investigate their interactions. The present short communication illustrates how to conduct multi-element X-ray movie imaging in a synchrotron facility solely by placing a micro-pinhole in front of a visible-light complementary metal-oxide semiconductor (CMOS) camera. It has been found that the CMOS camera can resolve X-ray fluorescence spectra when it is specially operated. In this work, a spatial resolution of ∼15 µm was achieved. In the X-ray movie, a movie frame acquisition time of 2 min and a spatial resolution of ∼50 µm were simultaneously achieved. It is clear that the CMOS camera can be a cost-efficient option for many researchers who wish to establish their own setup for visualizing chemical diffusion in various reactions.

## Abstract 2

A visible-light camera was used to resolve X-ray fluorescence spectra. Following the installation of a micro-pinhole, simultaneous multi-element X-ray fluorescence movie imaging was conducted in a synchrotron facility.

## 1. Introduction   

Movies are useful as a record of the overall progress of chemical reactions. Generally, a movie captured in visible light cannot reveal the elemental composition of a reaction. By contrast, X-ray fluorescence (XRF) can aid the identification of elements with quantitative information such as average concentration, and XRF imaging can reveal their spatial distribution. Therefore, a movie comprising a continuous series of XRF images can be a powerful tool for exploring multi-element reactions such as ion exchange, composition segregation, selective precipitation and formation of chemical-gradient materials (Zhao & Sakurai, 2017*a* ▸).

Full-field XRF imaging is an efficient way to collect XRF images. The technique requires an X-ray area detector that also possesses spectroscopic capability. Thus far, several options exist including a pn charge-coupled device (CCD) (Strüder *et al.*, 2001 ▸; Scharf *et al.*, 2011 ▸) and single-photon-counting CCD (Romano *et al.*, 2014 ▸, 2016 ▸; Zhao & Sakurai, 2017*b* ▸). The present short communication indicates the cost-efficient alternative of a visible-light complementary metal-oxide semiconductor (CMOS) camera used for direct detection of X-rays (Janesick, 2001 ▸; Holst & Lomheim, 2007 ▸). In previous work, it was found that the camera could resolve XRF spectra using laboratory X-ray sources (Zhao & Sakurai, 2017*c* ▸). Here, the camera operation is performed in a synchrotron facility and its multi-element movie imaging capability with potential for high spatial resolution is illustrated.

## 2. Experimental   

The visible-light CMOS camera (PCO.edge 5.5, PCO AG) used in the present work has 2560 × 2160 pixels with a pixel pitch of 6.5 µm × 6.5 µm. The sensor was electrically cooled down to 5°C. For full-field X-ray imaging, the optical window of the camera was replaced with a 25 µm-thick beryllium X-ray window. A pinhole was drilled onto 50 µm-thick tungsten foil, which was then installed in front of the X-ray window. To measure the X-ray photon energies, the camera was operated in single-photon-counting mode, and all camera images were processed using an integrated-filtering method (Zhao & Sakurai, 2017*c* ▸) for charge-sharing correction.

Experiments were conducted in the BL-14B Photon Factory of KEK, Japan. The primary X-ray beam passed through an Si(111) double-crystal monochromator and was collimated to a width of 1 mm and height of 10 mm. The energy used in the present work was 13.5 keV. In the imaging experiments, samples were positioned vertically with a glancing angle of approximately 5° between the primary X-ray beam and the sample surface (Fig. S1 of the supporting information).

Two additional modifications were necessary for applying the camera to synchrotron experiments. First, the top part of the camera housing was changed from aluminium to 2 mm-thick brass for shielding against high-energy X-ray scattering. Second, the double-crystal monochromator was detuned to eliminate higher-order harmonics that contribute strongly to noise collected by the camera sensor.

## 3. Results and discussion   

**Figure 1:**
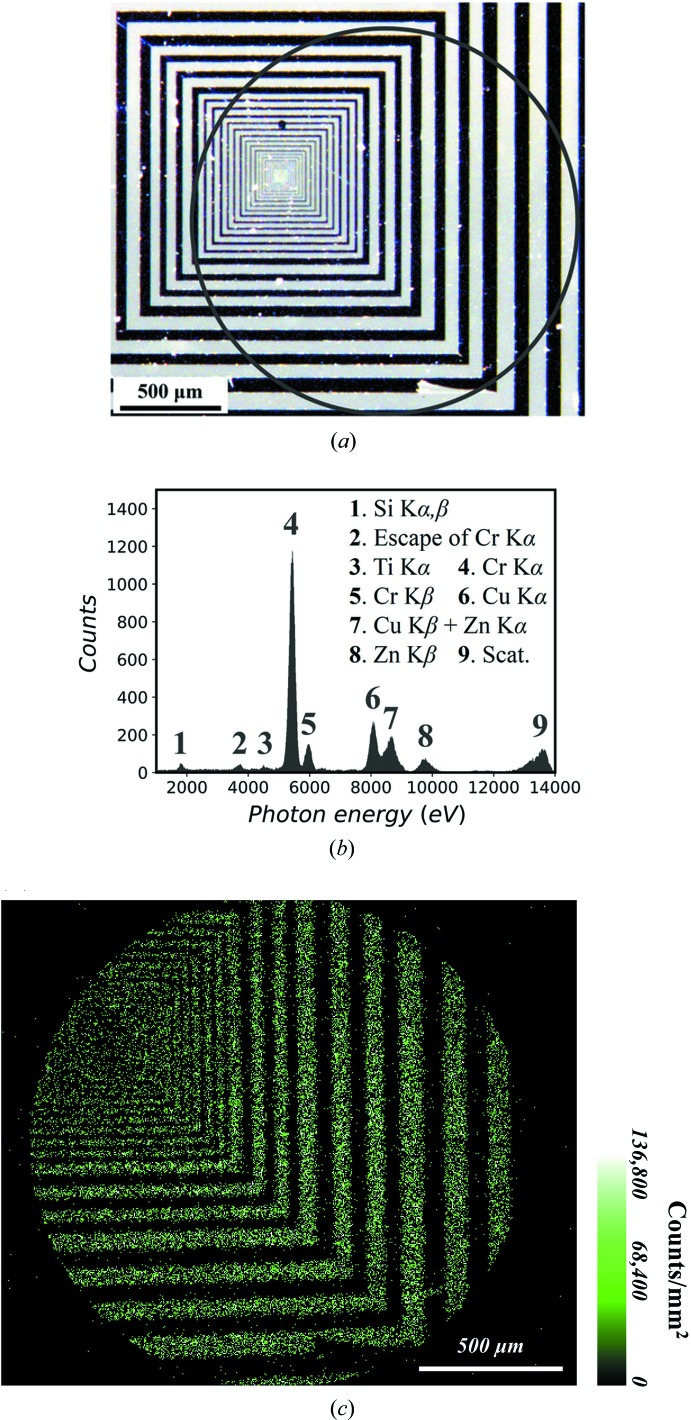
Full-field XRF imaging of a commercial-resolution target sample. (*a*) An optical photograph of the resolution target. The black rectangular patterns are chromium coatings. The XRF imaging area is enclosed by the circle and the scale bar is 500 µm. (*b*) Full-field XRF spectra accumulated for 6 h. (*c*) An XRF image of Cr *K*α and *K*β.

The spatial resolution of the full-field XRF imaging system was tested using a pinhole diameter of 10 µm. The distances from the pinhole to the target and camera sensor were 1.65 mm and 14 mm, respectively. The resolution target was a chromium pattern coated on silica glass [Fig. 1 ▸(*a*)]. The total measurement time was 6 h. Subsequent full-field XRF analysis [Fig. 1 ▸(*b*)] shows the spectral peaks of chromium from the target. XRF contamination of copper and zinc, which originated from the brass housing of the camera, was also observed. The XRF image of chromium [Fig. 1 ▸(*c*)] indicates a spatial resolution of 15 µm or even better for the system.

**Figure 2:**
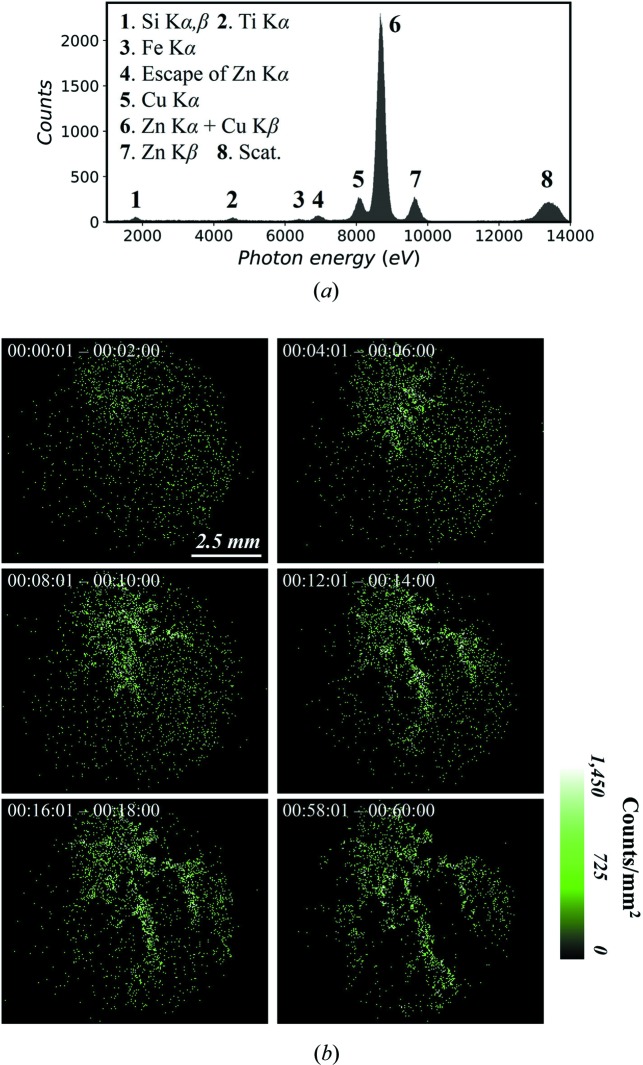
Application to observation of growing zinc electrodeposits. (*a*) Full-field XRF spectra. (*b*) Some key frames of the movie. The acquisition time for one frame is 2 min.

To demonstrate multi-element X-ray movie imaging, the growth process of zinc dendrites during electro-deposition was recorded. The reaction occurred in a thin container with dimensions of 30 mm (W) × 20 mm (L) × 1 mm (D). The container was filled with 0.1 *M* ZnCl_2_ solution, and two copper electrodes were immersed (Fig. S1). The distance from the upper tip of the cathode to the lower horizontal anode wire was 15 mm. The electric potential between them was 3 V. The front surface of the container was made of a 50 µm-thick polyester film to allow the X-rays to pass through. In this experiment, a 50 µm pinhole was used to collect a stronger XRF signal although this reduces the spatial resolution. The distances from the pinhole to the reaction cell and camera sensor were 8 mm and 14 mm, respectively. After connecting the circuit to initiate the reaction, the experiment continued for 1 h, as did the movie recording. In the full-field XRF spectra [Fig. 2 ▸(*a*)], most events in the Zn *K*α peak came from the reaction cell (not the brass shielding), and therefore the spectral imaging of Zn *K*α showed the zinc distribution in the reaction. In this way, images of zinc were generated every 2 min, and a continuous movie was obtained [Fig. 2 ▸(*b*)]. These XRF movie frames confirm the visual observation that zinc dendrites first appeared on the upper tip of the cathode and then gradually grew downward; they also show what visible light cannot: the zinc dispersed in the solution and was gradually exhausted.

In the spectra shown in Figs. 1 ▸(*b*) and 2 ▸(*a*), the small peak of Si *K* *α,β* originated from the Si-based camera sensor. The small peaks of Ti *K*α and Fe *K*α are assigned to trace environmental contaminants. Furthermore, in this work the XRF spectra have a slight spectral contamination of zinc and copper from the brass camera housing. In future, this contamination can be easily eliminated by optimizing the housing shape or coating other high-*Z* ‘XRF-silent’ elements. Meanwhile, the present work shows that the spatial resolution of full-field XRF imaging can reach 15 µm by using a pinhole of a sufficiently small size. When employing this simple approach, the XRF intensity collected by the pinhole becomes weaker as the pinhole becomes smaller. Consequently, a larger pinhole was adopted in the movie experiment as a compromise. To reduce this problem, a higher-flux synchrotron X-ray beam may be used to compensate the intensity loss, or the pinhole may be replaced by other X-ray imaging optics such as a collimator plate (Sakurai, 1999 ▸; Sakurai & Eba, 2003 ▸; Sakurai & Mizusawa, 2004 ▸; Mizusawa & Sakurai, 2004 ▸; Eba *et al.*, 2016 ▸). Other approaches explore the use of polycapillaries (Scharf *et al.*, 2011 ▸), modified uniformly redundant array masks (Haboub *et al.*, 2014 ▸) and potentially other devices in this context.

The silicon-based image sensor of the CMOS camera was estimated to be thinner than 10 µm by measuring and comparing its absorption efficiency for X-rays of different energies. Therefore, the visible-light CMOS camera has a lower detection efficiency for high-energy X-rays compared with many professional X-ray area detectors (Strüder *et al.*, 2001 ▸; Henrich *et al.*, 2009 ▸; Dinapoli *et al.*, 2011 ▸; Blaj *et al.*, 2016 ▸). However, its low cost and contemporary availability provides an excellent opportunity for many researchers to establish their own setups. Moreover, considering its unique advantages of a large pixel count and small pixel size, it is clear that the CMOS camera has potential as the XRF detector of choice for visualizing chemical diffusion in various reactions.

## Supplementary Material

Point-by-point responses to reviewers #1, #2 and #3, as well as the list of revisions. DOI: 10.1107/S1600577518014273/co5110sup1.pdf
