# Supplementary material for: Multi-element X-ray movie imaging with a visible-light CMOS camera
Source: J Synchrotron Radiat. 2019 Jan 1;26(Pt 1):230–3. doi: 10.1107/S1600577518014273 (PMC6337888; doi:10.1107/S1600577518014273)
Supplement: Supplementary file 1 [file s-26-00230-sup1.pdf]

JOURNAL OF SYNCHROTRON RADIATION

Volume 26 (2019)

Supporting information for article:

Multi-element X-ray movie imaging with a visible-light CMOS camera

Wenyang Zhao and Kenji Sakurai

Supporting Information

Multi-element X-ray movie imaging with a visible-light CMOS camera

Wenyang Zhao[ab] and Kenji Sakurai[ba*]

[a] *University of Tsukuba, 1-1-1, Tennodai, Tsukuba, Ibaraki, 305-0006, Japan*

[b] *National Institute for Materials Science, 1-2-1, Sengen, Tsukuba, Ibaraki, 305-0047, Japan*

Correspondence email: sakurai@yuhgiri.nims.go.jp

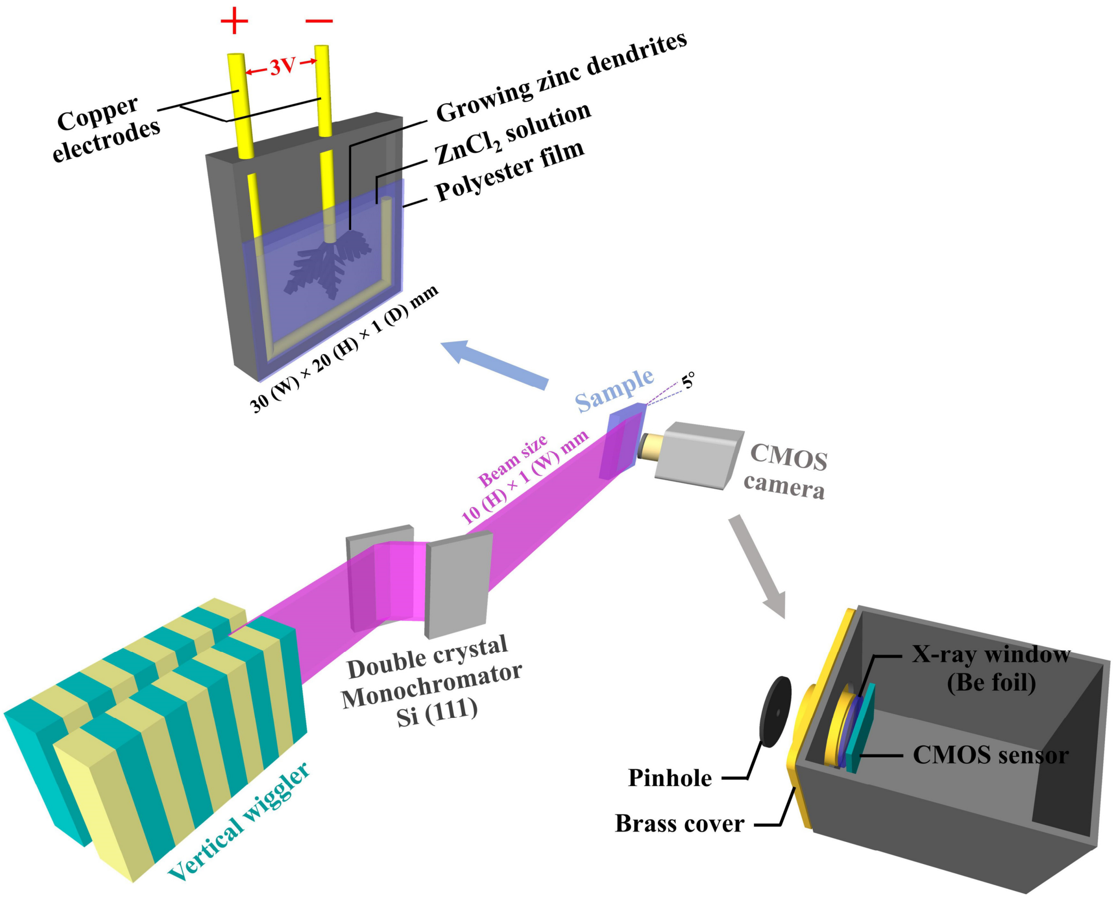

Figure S1 A schematic of the experimental setup. The experiments were conducted in BL-14B, Photon Factory, KEK, Japan. The beamline utilizes a vertical wiggler and a double-crystal monochromator in which the reflection plane is Si (111). Other components in the beam path are omitted in this schematic.
